# Popliteal artery thrombosis as a rare complication of paroxysmal nocturnal hemoglobinuria (PNH): A case report

**DOI:** 10.1016/j.ijscr.2021.106445

**Published:** 2021-09-24

**Authors:** Kiran Bhusal, Prashiddha B. Kadel, Khagendra Bhandari, Shova Aryal, Nitin Gyawali, Ashok Kushwaha, Kajan Raj Shrestha, Anjan Shrestha

**Affiliations:** a Maharajgunj Medical Campus, Institute of Medicine, Kathmandu, Nepal; b Manmohan Cardiothoracic Vascular and Transplant Center, Tribhuvan University, Kathmandu, Nepal; c Department of Hemato-Oncology, Tribhuvan University Teaching Hospital, Kathmandu, Nepal

**Keywords:** Paroxysmal nocturnal hemoglobinuria (PNH), Below knee amputation (BKA), Arterial thrombosis

## Abstract

**Introduction and importance:**

Popliteal artery thrombosis a rare but life-threatening complication of Paroxysmal Nocturnal Hemoglobinuria caused due to hemolysis. Complications of further thrombotic event are common after initial management.

**Case presentation:**

A 38 years old male, known case of PNH for 2 years, presented with the history of loss of pain sensation, coldness and loss of movement in left lower leg for 5 days and history of multiple blood transfusion. The patient underwent knee amputation because of possible complication of PNH.

**Clinical discussion:**

Intermittent claudication along with paresthesia, lower extremity weakness, stiffness, and cool extremities are seen in patients of Peripheral Artery Disease. Ultrasound color duplex is a sensitive and specific examination for peripheral flow while gold standard techniques like MRI and CT angiogram to detect and diagnose arterial lesions.

**Conclusion:**

The risk of thrombo-embolism in a patient of PNH should be considered by a treating doctor while early imaging and management should be done to reduce the complications and risk of amputation.

## Highlights

• Popliteal artery thrombosis is a rare life-threatening complication of Paroxysmal nocturnal hemoglobinuria (PNH)
• We report a known case of PNH patient diagnosed with acute occlusion of Popliteal artery due to thrombosis.
• Complications of further thrombotic event are common after initial management and subsequent above knee amputation was done.

## 1 Introduction

Paroxysmal nocturnal hemoglobinuria (PNH) is a rare bone marrow failure disorder that manifests with hemolytic anemia, thrombosis, and peripheral blood cytopenia with the absence of two glycosylphosphatidylinositol (GPI)-anchored proteins (CD55 and CD59), leading to the uncontrolled complement activation that accounts for hemolysis and other PNH manifestations [1]. Phosphatidylinositol Glycan Anchor Biosynthesis Class A (PIG-A) is a gene that participates in the early step of GPI anchor biosynthesis responsible for PNH. Here the affected granulocytes and B lymphocytes have the same somatic mutation of PIG-A, indicating their clonal origin from a multipotential hematopoietic stem cell [2]. Thrombosis is the main culprit of death in PNH, and an initial thrombotic event increases the relative risk of death by 5- to 10-folds [3]. Thromboembolism is mainly due to hemolysis, larger the PNH clones in patient greater is the chance of hemolysis and thromboembolism. Although the mechanism is not fully understood, hemolysis has been implicated in the initiation of platelet activation and aggregation [4]. This report has been written in line with SCARE guidelines [ 5].

## 2 Case presentation

**Fig. 1:**
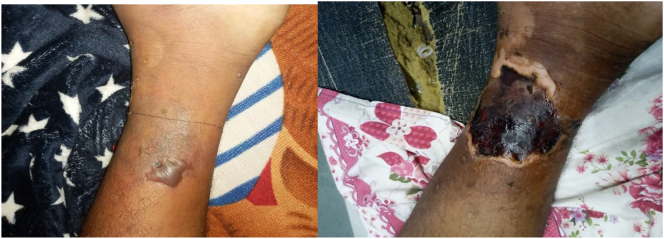
The progression of the limb ischemia due to arterial thrombosis is shown in the figure. The first figure shows the bulla in the medial aspect of the lower leg at the initial stage (A). The bullae ruptured and turned out to an ulcer with necrotic black base during presentation to our center (B).

A 38 years Hindu male, a known case of PNH, came to the outpatient department of Manmohan Cardiovascular and Transplant center with chief complaints of loss of pain sensation, coldness and loss of movement for 5 days in left lower leg. The patient also complained of ulcer in the medial aspect of the left lower limb for 2 months. Before presentation to our OPD, the patient had bulla in that area, which ruptured slowly and got converted to black ulcer (Fig. 1 a and b). There was hindrance in daily activities and movements like walking.

The patient was diagnosed as PNH 2 years back by flow cytometry to identify the GPI-AP deficient peripheral blood cells when he presented with progressive fatigue, weakness and fainting episodes. The patient was thus managed by medications and was under anticoagulants and iron supplementation and multiple blood transfusions in the past. The patient had no history of surgical intervention done. He didn't complain of fever, shortness of breath, chest pain, vomiting and edema. He had no chronic illness like Diabetes mellitus, Hypertension, Thyroid disorders and Tuberculosis. There was no family history of similar illness and trauma in the past. The patient was nonalcoholic and non-smoker. The color of urine was yellow.

On examination he looked well built, nutrition intake was adequate and on general examination, pallor was present, icterus was not present and his vitals were Blood pressure (BP) of 140/80 mm of Hg, Pulse rate 120 beats per minute, Respiratory rate 20 breath/min, Temperature 98-degree Fahrenheit and oxygen saturation of 98% in room atmosphere. Popliteal artery was palpable but dorsalis pedis was not palpable. On examination of his diseased leg, the motor, sensory and reflexes of the legs were lost. But, Respiratory system and cardiovascular system (CVS) were normal.

### 2.1 Investigations

On admission the patient had a hemoglobin level of 3.8 g/dL (14–18 g/dL) due to hemolysis, differential leukocyte count with Raised neutrophils 82% and decreased Lymphocyte 13% (25%–45%). Total RBC count was 1.1 million (4.5–5.5 million), PCV was 26.3% (36%–54%) and high MCV of 96.7% and his platelets count was 401,000/cumm (150000-400000cells/cumm) and prothrombin time 23 s (10–12 s) and PT INR 1.89 as he was put under warfarin therapy. His lactate dehydrogenase level was 1730 U/L (0–246 U/L) as a result of hemolysis. Serological tests done were all negative. His sodium was 134 mEq/L (135–146) and potassium of 2.9 mEq/L (3.5–5.2) which were slightly deranged likely due to fluid imbalance.

Liver functions test shows slight derangement. Total bilirubin 1.4 mg/dL (0.3–1.2), direct bilirubin 0.4 mg/dL (<0.2). Alkaline phosphatase 244 U/L (30−120), SGPT 59 U/L (<50), SGOT 52 U/L (<50).

**Fig. 2:**
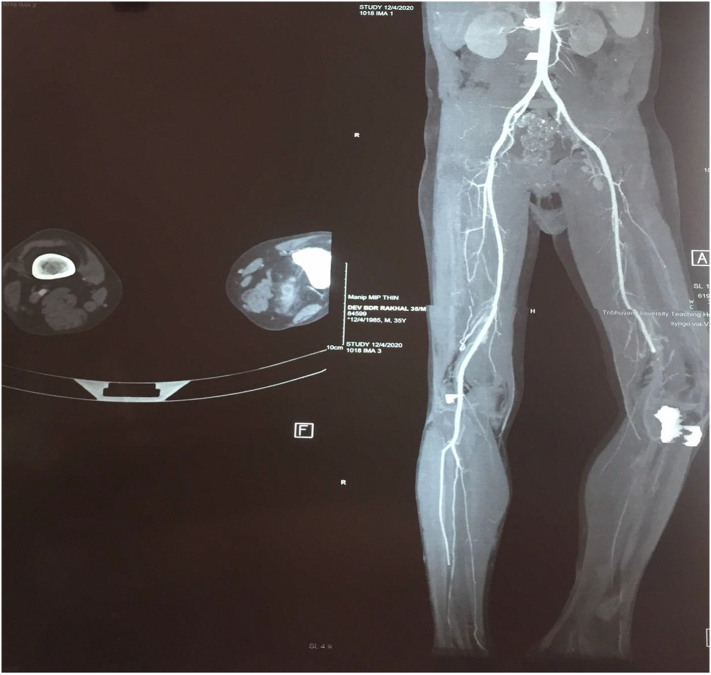
CT angiography in the left leg showing contrast opacification of the distal superficial femoral artery with abrupt cutoff at the origin of popliteal artery. Distally faint reformation of the anterior and posterior tibial artery present.

In suspicion of thrombosis, arterial study of B/L lower limb was done which showed arterial thrombosis of left popliteal artery and tibio-peroneal artery with absence of flow in left posterior tibial artery along with thickened and edematous skin and subcutaneous tissue in anterior-medial aspect of lower one third of left leg. Though the ultimate specific treatment of ALI grade III is amputation, there was no life threating condition like sepsis related ALI. In order to prevent further complication and future embolization we planned for CTA urgently before surgery. The details finding of CTA is shown in (Fig. 2).

### 2.2 Differential diagnosis

After exclusion of common differentials, we came to the conclusion that the left lower limb ischemia (Grade III) because of popliteal artery occlusion due to acute occlusion of popliteal artery. A hemato-oncologist consultation was also done for the possible progression and complication of PNH.

### 2.3 Treatment

**Fig. 3:**
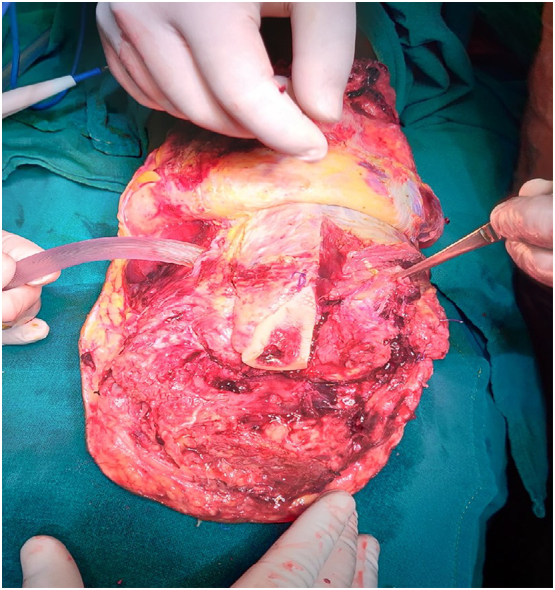
Intraoperative picture of below knee amputation (BKA). The stump of below knee amputation with Yankur suction and forceps. The bone visualized is Tibia.

Initially, the wound was managed conservatively and discharged at the local center. The dressing and debridement were done in that center hoping for the wound to heal. He was not presented to higher center before the gangrene had appeared. But within 2 months, he presented in our emergency with acute limb ischemia (Grade 3) of left lower leg and as the condition was irreversible and no role of revascularization. At first, the embolization was done and the proximal flow was good enough with adequate collateral. Our plan initially was to perform AKA but considering patient age and as he was the main worker of his family and patient preference to use prosthetic legs. Also, we found adequate collateral around the stump. So, we went for below knee amputation. The operation was performed by the team of Vascular surgeon of this center. The intraoperative picture of the Below Knee amputation (BKA) is shown in the Fig. 3.

Post operatively, the patient was managed with IV antibiotics and analgesics. Daily dressing was done. After 10 days of the stay, the patient was stable, wheelchair bound and discharged. He was advised to follow up after one week. The patient was well after the post-operative period, was thriving and satisfied with treatment but depressed with his own condition.

### 2.4 Outcome and follow up

**Fig. 4:**
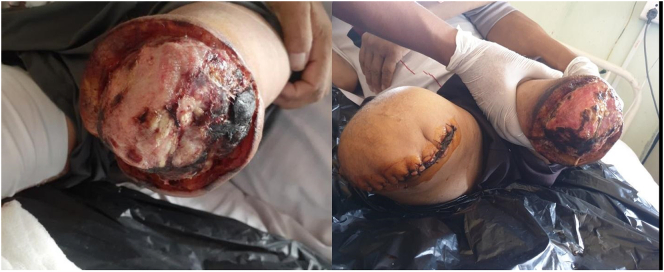
The above knee amputation of the Leg after his second admission and third visit. The bilateral above knee amputation is shown is the right side of figure following the left at the present.

During the discharge he was within therapeutic INR of 2.4. Despite the anticoagulation therapy after BKA, a patient presented late with gangrene of the amputation stump on follow up after 1 and half month and his INR was deranged because he missed his timely follow up. A revision amputation was planned. During the second hospital admission, the patient underwent above knee amputation of left limb. The condition of leg and wound is shown in Fig. 4.

**Fig. 5:**
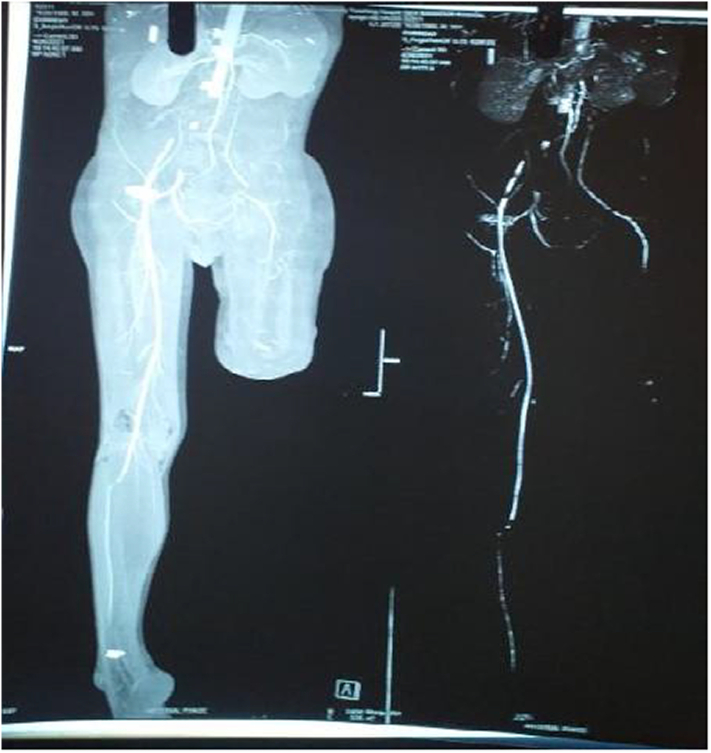
Demonstration of abrupt cut-off at bifurcation of common iliac artery with distal reformation of right and left superficial femoral artery. The right superficial femoral artery along its course after giving rise to popliteal artery fails to give rise to tibioperoneal trunk just below the knee.

In third visit, after 15 days the patient came with features of acute limb ischemia (Grade III) of the right lower limb. CT angiogram of the thoracic, abdominal aorta and bilateral lower limb revealed complete obstruction of bilateral common femoral artery (Fig. 5). As the patient underwent no recovery and had severe pain with non-salvageable limb. So, above knee amputation of right leg was done to further reduce the complications. The bilateral above knee stump is shown in the figure. The patient tolerated the treatment but severe pain existed. The patient was wheelchair bound with disability. The pain was managed conservatively and anticoagulants were continued. The patient was advised for constant hemato-oncology follow up.

## 3 Clinical discussion

PNH is a rare disease with the prevalence from 12 to 13 per 1,000,000. The incidence rate over the study period was 5.7 per 1,000,000 person-years [6]. Thrombosis is the leading cause of death, and an initial thrombotic event increases the relative risk of death by 5- to 10-fold in PNH [3].

Beneficial effect of eculizumab on thrombotic risk suggests a major role for complements activation. Also, the deficiency of GPI-anchored proteins involved in hemostasis may be implicated but the mechanism of thrombosis in PNH is still unknown. Thrombosis in PNH results in high morbidity and mortality. Thrombosis often occurs at unusual locations, with the Budd–Chiari syndrome being the most frequent manifestation. Vitamin K antagonists reduce the risk but do not completely prevent thrombosis. Other most common sites include the intra-abdominal and cerebral veins [7].

The arterial thrombosis of the popliteal artery in our case is rare. Men and women have typical, atypical, or asymptomatic Peripheral Arterial Disease (PAD) and studies have shown that the majority of PAD patients do not have typical claudication [8], [9]. Intermittent claudication is the most classic symptom of PAD characterized by an exercise-induced cramping sensation with associated fatigue, weakness, and or pressure exacerbated by leg elevation and relieved by placing the limb in a dependent position. Paresthesia, lower extremity weakness, stiffness, and cool extremities may also be present. 70% to 80% of patients have stable intermittent claudication over 10 years; however, a portion of patients may progress through debilitating ischemic rest pain, critical limb ischemia, and eventual amputation [10]. Our patient also presented with above symptoms.

In literature, only few case reports have described about the relation of limb ischemia and PNH. PNH patients have predisposition for both arterial and venous thrombosis and hypercoagulable state even after standard anticoagulant. Crawfold et al. described a 65-year-old woman with known PNH and peripheral arterial disease who presented with critical limb ischemia and a nonhealing left foot ulcer; later underwent above knee amputation similar to our case [11].

The diagnosis and detection of chronic limb ischemia is the holistic approach requiring the identification of cardiovascular risk factors and the evaluation of peripheral pulses [12].Ultrasound color duplex is a sensitive and specific examination for peripheral flow [13], [14]. MRI and CT angiogram are the gold-standard imaging to detect the arterial lesions as they provide an accurate description of obstructions and help the vascular surgeons or the interventional radiologist performing the revascularization [15]. In our case, CT angiogram was done to describe the artery thrombosis.

The incidence of peripheral arterial embolism has decreased due to wider use of anticoagulation and decreasing rates of RHD [16]. the introduction of the Fogarty Embolectomy Catheter in 1963 allowed minimally invasive technique and local anesthesia to be used for embolectomy and standard treatment in present [17]. Complicated techniques such as arterial angioplasty, thrombolysis and possibly arterial bypass are the treatments for more complicated arterial thrombosis [16].Ischemia of the leg for more than 12 h may lead to the chance of amputation [18].Long-term therapy after immediate reperfusion is dictated by the origin of the embolism i.e., long-term aspirin or clopidogrel therapy is also recommended [19]. Trauma and traditional bone setter gangrene are the commonest indications for leg amputations. The commonest complication is wound infection with identifiable predisposing factors. Thus, paying attention to predisposing factors can reduce complications [20]. Our patient had prolonged ischemia of leg for more than 12 h with complications in wounds like gangrene opted for amputation.

There is significant mortality and morbidity following major amputations of leg. Survivors with BKA require revision or conversion to AKA infrequently [21]. A successful below knee amputation (BKA) of left lower limb secondary to PNH was done in this case without any complications.

## 4 Conclusion

Thrombosis of the peripheral arteries should be considered as the possible complication of PNH beside the most common visceral artery thrombosis. Early recognition of symptoms like Pain, intermittent claudication, Coldness of extremities and paresthesia should make vascular surgeons and hematologists treating the PNH aware of peripheral artery thrombosis. Complications of further thrombotic event are common after initial management. CT angiography and Doppler should be performed while early and appropriate treatment should be done. The patient could have been managed medically if the Acute limb ischemia could have been presented to tertiary center as early as possible but due to delay, he is disable now after AKA so, it is necessary to provide training to the primary health care worker about its detection and timely referral to higher centers in a developing country like Nepal.

## Funding

This research did not receive any specific grant from funding agencies in the public, commercial, or not-for-profit sectors.

## Ethical approval

Nothing to declare.

## Informed consent

Written-informed consent was obtained from the parent of the patient for publication of this case report and the images. A copy of the written consent is available for review by the Editor-in-Chief of this journal on request.

## Authors contribution

**SA, AS and KB** = Study concept, Data collection.

SA, and KB* = Writing- original draft preparation.

**PBK and AK** = Editing and writing.

**KRS and PBK** = Proofreading and Editing and surgical therapy for the patient.

**AS** = Anjan Shrestha**, KB**=Kiran Bhusal, KB* = Khagendra Bhandari, **SA** = Shova Aryal, **KRS** = Kajan Raj Shrestha, **PBK** = Prashiddha B Kadel.

All the authors read and approved the final manuscript.

## Research registration

Not applicable.

## Note

No patient or author details are included in the figures.

## Guarantor

Prashiddha B Kadel accept full responsibility for the work and/or the conduct of the study, had access to the data, and controlled the decision to publish.

## Provenance and peer review

Not commissioned, externally peer-reviewed.

## Declaration of competing interest

None to declare.
